# A Framework for the Specificity of Addictions

**DOI:** 10.3390/ijerph8083399

**Published:** 2011-08-18

**Authors:** Steve Sussman, Adam Leventhal, Ricky N. Bluthenthal, Marilyn Freimuth, Myriam Forster, Susan L. Ames

**Affiliations:** 1 Departments of Preventive Medicine and Psychology, University of Southern California, California 90032, CA, USA; E-Mails: adam.leventhal@usc.edu (A.L.); rbluthen@usc.edu (R.N.B.); myriamforster@msn.com (M.F.); 2 Clinical Psychology, Fielding Graduate University, Santa Barbara, California 93105, CA, USA; E-Mail: mfreimuth@fielding.edu; 3 School of Community and Global Health, Claremont Graduate University, Claremont, California 91711, CA, USA; E-Mail: susan.ames@cgu.edu

**Keywords:** addiction specificity, PACE model

## Abstract

Research over the last two decades suggests that a wide range of substance and behavioral addictions may serve similar functions. Yet, co-occurrence of addictions has only been reported among a minority of addicts. “Addiction specificity” pertains to a phenomenon in which one pattern of addictive behaviors may be acquired whereas another is not. This paper presents the PACE model as a framework which might help explain addiction specificity. Pragmatics, attraction, communication, and expectation (PACE) variables are described, which may help give some direction to future research needs in this arena.

## 1. Introduction: Addiction as a Biopsychosocial Phenomenon Involving a Range of Different Behaviors

For many years, researchers and practitioners have discussed various seemingly irrational behaviors that exhibit patterns of self-destruction similar to drug abuse [1–8]. Indeed, the concept of “addiction” has broadened in scope from referring to only physiologic processes related to drug misuse (pharmacodynamic tolerance and withdrawal) to a more elaborate biopsychosocial syndrome with commonalities across several behaviors. An overarching feature of the addictive process includes compulsively performing a behavior, for example, continuous drug taking, binge eating, gambling or working [2,4,9–14].

During the “addictive process” [2,4,5] initially one may pursue some course of action for appetitive effects such as pain reduction, affect enhancement, arousal manipulation, or fantasy. Over repeated engagement in the behavior, the individual becomes intensely preoccupied with the behavior despite diminishing appetitive effects [15,16]. Subsequently, the individual, if desiring to control or stop the behavior, experiences subjective loss control over when the behavior is initiated, how it is manifested, or when it will stop. Finally, one incurs negative consequences (e.g., social, role, physical, emotional) while continuing to engage in the self-defeating behavior. Stopping the behavior becomes difficult for several reasons, including having a lack of awareness of the “stimuli” or triggers that influence the behavior and the cognitive salience of immediate gratification relative to delayed adverse effects. That is, the behavior becomes increasingly more automatic and less under one’s control-ability [17–20]. At this point, the individual also may fear having to cope with day-to-day perceived stress and other life experiences upon cessation (possibly due to accumulation of addiction-related consequences, or having to endure “raw” emotional experiences without concurrent self-medication [5]), as well as having to suffer withdrawal-related phenomena [4,11,14]). Various substance and process/behavioral addictions appear to be intricately connected in terms of etiology, patterns of behavior, and consequences [12,21].

### 1.1. Patterns of Addiction Co-Occurrence

While it is not entirely clear what differentiates addictive-prone from non-addictive prone behaviors [22], Sussman, Lisha and Griffiths [14] identified 11 relatively common behaviors that appear to have addiction propensity (tobacco use, alcohol use, illicit drug use, binge eating, gambling, internet use, love, sex, exercise, work, and shopping). That article reported the prevalence and cooccurrence of these behaviors based on a systematic review of the literature. Data from 83 studies (each study *n* equal to or greater than 500 subjects) was presented and supplemented with smaller-scale data. The authors noted a 23% average co-occurrence among the 11 addictions (with a range from 10% to 50% overlap among 110 pairs of co-occurrence examined), and determined that approximately 50% of the U.S. adult population has suffered from signs of some type of addictive behavior over a 12-month period, controlling for co-occurrence. Although there are some methodological limitations [14], their findings suggest that there is marked variability in the pattern of addiction co-occurrence. As examples, addictions to cigarettes, alcohol, and illicit drugs are highly associated with each other and with sex and love addiction, which also are highly associated with each other. In addition, gambling addiction is strongly associated with cigarette smoking addiction but not as much with illicit drug abuse or dependence. Exercise addiction is moderately associated with eating, gambling, work, and shopping addictions but is more weakly associated with cigarette, alcohol, and illicit drug addictions [14].

One approach to classifying patterns of addiction co-occurrence purports three categories of individuals: (1) people who experience multiple addictions concurrently [11,14,23,24]; (2) people who experience substitute addictions; that is, where one addiction takes the place of a previously terminated addictive behavior in order to serve the same functions [11,25]; and (3) people who experience only one addiction in their lifetime [14]. However, even within the broad categories of multiple and substitute addictions, there is likely to be considerable differentiation; that is, individuals differ in the functions of addictions they suffer. For example, as was found among a sample of 543 mostly adult consecutive admissions to an addictions treatment center, addiction clusters appear to divide most generally into “hedonistic” (excitement/dominance motives, such as drug use, sex, love/relationship, gambling) and “nurturance” (providing for self or others motives, such as food, shopping, work, exercise) types of addictions [23].

Also, there has been some focus in the literature towards identifying the factors that explain a tendency for some individuals to develop co-occurring addictions [12,21,23,26,27]. For example, Carnes, Murray and Charpentier [21] presented ten different models of co-occurring addictions that they labeled “Addiction Interaction Disorder,” based on self-reported experiences of 1604 adult sex addicts. They identified several processes that account for co-occurring addictions, such as “cross tolerance” and “masking.” Cross tolerance occurs when one addiction causes a pre-existing tolerance to a second addiction such that the effects of the new addiction are dampened. “Masking” is where one addiction provides an alibi for another addiction (e.g., such as getting drunk prior to engaging in anonymous or casual sex). By contrast there has been relatively little attention directed towards explaining why some individuals are prone to some addictions, but not others (*i.e.*, addiction specificity).

### 1.2. Addiction Specificity and the PACE Model

A concept that pertains to why some addictions may not co-occur within individuals may be labeled as “addiction specificity.” Addiction specificity is a phenomenon complementary to addiction co-occurrence. Overall, different people appear to show unique patterns of addiction and, while they struggle with one or more addictive behaviors, they may not have difficulty with other potentially addictive behaviors. There are people, for example, who develop problems with drugs and sexual behavior but who never experienced difficulties with gambling (e.g., never lose much money when gambling, do not gamble long hours or lose control of their gambling behavior.) The epidemiology of addiction specificity across substance and process addictions has not been conclusively quantified, though some good work on addiction clusters is now being examined and may pertain to addiction specificity as well as co-occurrence, see [23]. As previously mentioned, Sussman, Lisha, and Griffiths’ [14] review on addiction co-occurrence provides emerging support that patterns of addiction specificity are relatively prevalent.

To date, no one model has been utilized to explain addiction specificity that considers the interplay between biological, environmental, situational, and learning factors. Although any individual may be susceptible to developing an addiction, it is unlikely that one’s genetic profile alone could determine specificity of addiction. Nevertheless, genes influence neurobiological systems and, in turn, responses to reinforcers, and play a role in susceptibility to various appetitive behaviors, see [1,3,28,29]. For example, an individual may inherit a susceptibility to feel shy and then feel much better in social situations while engaging in some appetitive behavior, like alcohol use, possibly leading eventually to alcoholism [30]. However, acquisition of specific addictions *versus* others also involves exposure to unique social environmental experiences, and associative learning and memory processes resulting from those experiences, possibly leading to different behavioral phenotypes [18,31–33]. A comprehensive model of addiction specificity should consider the interplay among these variables, all which impact one’s tendency toward one addiction or set of addictions *versus* another.

**Figure 1.**
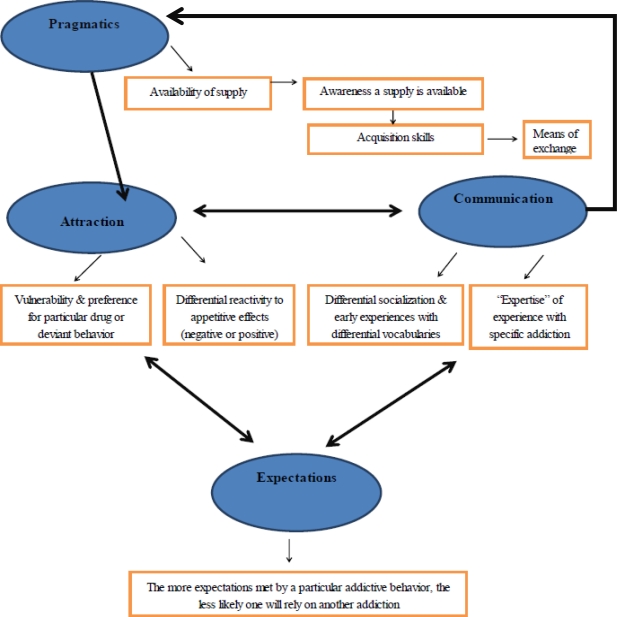
Diagram of the PACE model.

The PACE model was originally proposed by the lead author to explain the development of intimacy in relationships between two people from within the interpersonal attraction literature in social psychology [e.g., 34]. The variables that were formed from the interpersonal attraction/relationship development literature include residential propinquity and other practical variables which permit access to relationship development (“pragmatics”); physical attractiveness, speech tone, and personal habits which comprise how initially appealing persons will be to each other (“attraction”); sharing a common understanding of each other and of everyday experience (“communication”); and having cooperative expectations of each other (“expectation” [34]). Later on, the model was adapted and published as a means to describe the development of a relationship of a person with drug use [35] and, indeed, there are several similarities between the development of an entrenchment in an addictive behavior and the development of an intimate relationship [36]. As detailed below, this model provides a useful framework for understanding general processes that underlie specificity in the initiation and maintenance of addictive behaviors. Figure 1 depicts the current conceptualization of the PACE model.

## 2. Results and Discussion

### 2.1. Pragmatics

Pragmatics variables operate to discern whether or not one can access a particular addictive behavior and then engage in this behavior regularly. Pragmatics involves four aspects. First, there must be a *supply* of the object of the addiction available in the environment (e.g., drug distribution point, gambling casino, brothel, potential love partner, workplace, gym). If not, no relationship with the “addiction object [behavior]” can develop. Case in point, addiction to the internet was not possible prior to wide availability of the internet [14]. Objects of addiction tend to be available along distribution routes, which permit easiest passage from a manufacture/product/service origin point and where (consequently) there tends to be higher consumer demand [8]. Changes in availability of an addiction object can increase or decrease prevalence of addictive behavior. At a macro-geographical level, the explosion of crack cocaine use in the late 1980s in the United States or decline in heroin supply and use in Australia and the west coast of Canada are but two examples of this common phenomenon [37–39]. At a micro-geographical level, distance from an addiction source or supply is associated with overall prevalence of the behavior as well as disordered forms of the behavior (e.g., regarding alcohol use and abuse [40]; regarding gambling and problem gambling [41]). Nevertheless, if the addiction object is available, then other pragmatics aspects must be considered.

Second, one needs to be *aware* that there is a supply of the addiction object [service] available. In fact, perceived availability of the addiction object may be a more important predictor of behavior than objective measures of availability [40]. Promotion of the addiction object reaches the potential consumer by way of any number of channels (e.g., word of mouth, observation of sales, public venues such as clubs or bars, television advertisements, provider web sites, or even early evening news stories). “Channels of introduction” to the addictive behavior likely contain cues specific to that behavior and begin a process of differential exposure to and learning of information related to the context of the addiction [42], this perhaps being the earliest aspect of addiction specificity. For example, beer advertisements and packaging may indicate where to purchase the product, suggest that when one drinks beer one drinks multiple beers on a drinking occasion, and suggest how much of the addiction object to purchase [8].

Third, an individual must have *acquisition skills*; that is, one needs to know how to obtain the addiction object from the source. An individual needs to be able to converse appropriately with people who possess the addiction object (e.g., drug, sex), how to bring up topics without being threatening (e.g., cost, location, type of service), and how to arrange an exchange (usually money for the object). Finally, an individual needs to have a *means of exchange*; that is, possess money or services to offer in return for the addiction object. For example, one can pay for a drug, provide a service as a drug transporter, or offer sexual favors, as means to procure one’s drug of choice.

The means by which pragmatics influence addiction specificity are relatively intuitive—if the pragmatic variables are favorable to a trial of a potentially addictive behavior, the behavior is more likely to be initiated. The few current statements on specificity of addiction emphasize the importance of access and exposure to the addiction [12,27]. Of course, for some objects of addiction, such as food/binge eating, the pragmatics involved may render the behavior as a relatively easy one in which to engage, whereas some objects of addiction, such as heroin use, may be a relatively difficult one in which to engage [38,39]. However, many people have tried a variety of objects of addiction at least once. For example, by 12th grade 72% of youth in the U.S. have tried alcohol (55% have reported ever being drunk), 45% have tried cigarette smoking, and 47% of youth have tried an illicit drug (25% have tried an illicit drug other than marijuana [43]). A vast majority of the U.S. adult population (over 86%) have tried gambling at some time in their lives [44]. Most people have purchased shopping items “on impulse,” roamed the internet for a substantial amount of time, and looked at an erotic photo.

Situational opportunity and curiosity predict that a particular addictive behavior will be engaged in at least once. However, it is doubtful that pragmatics *per se* is the critical factor that leads to addiction specificity, particularly if multiple channels of addiction are readily available. It is likely that other processes are critical in channeling the transition from initiation of behavior to escalation, maintenance, and excessive or compulsive engagement in a specific addictive behavior or set of behaviors.

### 2.2. Attraction

Attraction plays an important role in addiction specificity by impacting whether someone is likely to initiate and then continue engaging in an addictive behavior. Numerous variables can shape what determines if a behavior is attractive. These include individual difference variables that may influence selection of the addictive behavior. For example, some addictive behaviors (e.g., heroin, involving needle use) may be more normatively stigmatized [45] and, hence, less attractive to many persons. However, those relatively vulnerable to engage in such behavior may prefer relatively stigmatizing addictive behaviors as a *prima facie* expression of defiance [14,27]. More specifically, persons attracted to relatively stigmatized behaviors such as heroin injection may initially intensely enjoy the reputation they obtain (e.g., deviant peer group credibility), or the reactions to their behavior that they observe from others, as being beyond the chains of social restraint, expressed in the addiction [45,46]. These individuals also may be less attracted to addictive behaviors that are more socially acceptable (e.g., shopping, internet). Conversely, those who are attracted to relatively deviant addictive behaviors may be interested in engaging in relatively deviant manifestations of other addictions. As examples, they may favor shoplifting as a form of shopping addiction or may become a workaholic sex worker (*i.e.*, work long hours at a relatively “extreme” job). Of course, those individuals who are relatively less enticed by deviance might be attracted to fewer types of addictive behaviors [1,3,47].

Individual differences in the initial acute reinforcing effects of addictive behaviors can shape one’s attraction to these behaviors [1,3,15,16,48,49]. Indeed, there is marked between-person variability in the acute effects of a variety of addictive behaviors [50,51]. That is, for some individuals a behavior can result in extremely pleasurable experiences (e.g., high, rush, relaxation, stimulation, social and performance enhancement). For others the same behavior can result in severe aversive effects (e.g., anxiety, undersired sedation, social and performance impairment, dysphoria), or relatively few or weak acute effects (neither positive nor negative). For example, some East Asians have a gene variant that produces an enzyme that inadequately breaks down alcohol’s initial metabolite, aldehyde dehydrogenase, and hence, they tend to experience uncomfortable physiological reactions such as a flushing response, nausea, and headaches when drinking alcohol. Thus, East Asians may be less attracted to using alcohol in comparison to other substances, such as marijuana or nicotine [27].

Certain intrapersonal traits may impact initial sensitivity to specific addictive behaviors. Anhedonia—the incapacity to experience pleasure in response to natural rewards—is unlikely to increase propensity for behavioral/process addictions that produce relatively less positive reinforcing effects (e.g., sex, shopping [52]). By contrast, anhedonia is associated with increased sensitivity to the euphorogenic effects of stimulant drugs (e.g., amphetamine and cocaine [53,54]). This variation in enjoyment of different drugs or of other addictive behaviors may be similar to notions about using drugs as a means of self-medication [55,56] or for satisfying a biologically-based desire for stimulation as in sensation seeking [57,58].

Attraction also involves the experiential pleasantness ascribed to addictive behavior-related stimuli and context. That is, one may feel attracted to the sight, smells, sounds, tactile stimulation, or social stimuli inherent in the context of the addiction [10]. For example, researchers and practitioners have noted that drug addicts appear to become addicted to the routine of preparing and administering the drug, and contextual cues associated with the drug [6]. Overtime, and through associative learning and memory processes, contextual stimuli may come to represent appetitive effects associated with the behavior [18,33,59], affecting attraction to the behavior itself. Interestingly, accidental circumstances may lead to avoidance of or preference for that addictive behavior [60]. For example, acting in a highly shameful way or experiencing pain following an accidental fall while using marijuana for the first time may cue one to avoid its use, though not the use of other drugs. It is important to note that external cues for an addictive behavior may be unique to that addictive behavior, and, hence, related behavior-specific urges would be elicited in response to those external cues as well as the addiction object [6,61].

In some instances, the shaping of addiction specificity may involve extended access and involvement with a particular addictive behavior during a critical point in childhood or adolescence which may facilitate an intense attraction toward the behavior. Neural adaptations may be especially likely when one is most neurobiologically vulnerable during adolescence. It is at this time that there exists relatively few higher-level inhibitory functions monitoring relatively greater motivational drive for novel experience and this may affect the course of an addictive behavior [62]. During adolescence some subcortical structures mature earlier and are more able to support the acquisition of appetitivetype behaviors [63,64]. Thus, the behavior may be maintained by early maturation of brain structures able to support the behavior without executive inhibitory control processes overriding the behavioral tendencies [20].

Between-person differences in disliking cessation of an addictive behavior also may be important for explaining the specificity in whether or not one maintains an addictive behavior after a habitual pattern is already established. For example, there are reports of marked individual differences in the severity of withdrawal symptoms following discontinuation of an addictive behavior [65]. It is possible that an individual has a greater propensity to experience severe withdrawal after abstaining from one addictive behavior compared to another behavior [66]. In this case, he or she is likely to continue one type of behavioral pattern to avoid severe withdrawal, and as a result, manifests addiction specificity.

### 2.3. Communication

People tend to select social and physical environments that are similar to earlier experienced environments, which may shape life experiences in part by repetition of learned patterns of communication (e.g., Life Course Theory [67]). For example, youth who early-on have learned to express anger-related words or cuss words are relatively likely to expose themselves to persons and situations that involve risky behaviors including addictive behaviors such as drug misuse, gambling, or sexual behavior [8,68]. Further, it is possible that earlier life experiences, by perpetuating differential communications associated with addictive behavior, may prepare people for which types of addictions they pursue [42]. That is, early experiences with differential vocabularies can direct behavior toward specific addictive behaviors. For example, observing older siblings engaging in marijuana use may teach one the language associated with marijuana use (e.g., lighters, matches, bongs, rolling papers, pipes, or head highs *versus* body highs, inhaling), preparing one for how to use marijuana when one is older [69,70]. At the same time, if one does not learn the language associated with another addictive behavior and, hence, does not tend to think in terms of the language of the other addictive behavior (e.g., gambling addiction: bet, action, call, payout, all-in, ante, an arm, wad; [71]), then communication becomes engrained specific to one addiction (marijuana) but not another (gambling).

There are several avenues by which communication processes may contribute to addiction specificity. Some people may originate from cultural backgrounds that cause them to feel comfortable or uncomfortable with taking part in the communication processes pertaining to a particular addiction, or lead them to be potentially unaware of words associated with the addiction. For example, Latter Day Saint or Baptist church members tend to avoid alcoholism or tobacco addiction and, in general, may be relatively likely to avoid discussion of these drugs [72].

As one continues to engage in an addictive behavior, a relationship develops that involves seeking, experiencing, and recovering from the effects of the addictive behavior. A system of communication about these aspects of the addiction may develop, encompass important features of one’s daily life, and call upon quite distinct personal and intergroup communication styles and techniques. For example, buying a drink in a bar requires different interpersonal communication skills than purchasing an ounce of cocaine from a dealer. The interactions among drinkers occur within the continuum of accepted social practices where both distributor and consumer often operate within the law (depending on other variables such as if the customer is “cut off” at some point in drinking, drugs are permitted at a bar, or whether drinking and driving are involved). In contrast, cocaine use may place users in jeopardy of physical aggression or theft from peers, and both users and dealers may incur legal consequences, facilitating perhaps a different interactional style including “code words” to arrange a buy (e.g., someone may request buying a “cup of soup” to indicate one “rock” of crack [73]). In general, “insider speech” may develop to serve as a symbol of commonality and group identification pertaining to specific addictions within specific contexts [74].

As one becomes differentially socialized, one may become an “expert” in the language of the addiction and feel like a “regular” or someone who belongs in that context. One may comprehend addictive behavior-specific words that associate the behavior with life experiences and show an understanding of the language of the behavior (e.g., “4:20” is jargon that refers to marijuana use in the United States by many experienced users: the time of day to use, marijuana appreciation day; “hand release” refers to a sex worker bringing a client to orgasm by using a hand, whereas “half and half” refers to engagement in a combination of oral and vaginal sex). Interaction with agents of an addiction (e.g., card dealers, sex workers) or other addicts becomes embedded with a commonality of terms that refer to the behaviors, associated objects or paraphernalia, or subjective experience. The person may self-identify with addiction-related groups or activities (e.g., running clubs, pertaining to exercise addicts). Additionally, individuals with one addiction may communicate disparagingly about another addiction. For example, some methamphetamine users may operate within social contexts that ridicule people who drink alcohol or engage in other behaviors that are sedating or result in certain types of performance impairment (e.g., slurring words). Communication about the addiction, therefore, can be a way of forming or solidifying exclusive social relationships with other addicts or addictive object providers [75].

### 2.4. Expectations

Various conceptualizations of the expectancy construct have been applied to research on addictive behaviors since Rotter [76] initially proposed expectancy theory. Expectancy as a construct relevant to addiction involves the anticipated consequences of behavior or beliefs held about the likelihood of appetitive effects [77–80]. In general, expectancies are subjective probabilities regarding the likelihood of achieving various outcomes by engaging in some behavior. In terms of the PACE model, addiction expectancies or expectations are beliefs regarding the likelihood that or extent to which an addictive behavior is providing solutions to experiential requests. One may expect or anticipate that the addictive behavior will provide specific outcomes such as helping one live life more comfortably in the immediate present (e.g., to lift self-esteem, complement well other daily activities, or provide a social lubricant effect [7,26,81]).

There are several factors that contribute to development of specific expectancies for particular addictive behaviors. These include one’s genetically inherited sensitivity to the behavior [82], emotional disposition (e.g., individuals with social anxiety tend to hold expectancies that alcohol facilitates social performance [83]), or motivational state (e.g., those with weight concerns may hold positive expectations regarding the appetite suppressing effects of tobacco [84]). Importantly, though, specific expectancies develop through the interplay of individual difference variables with vicarious social learning, as well as with direct experience. For example, hearing comments relevant to expectancies for reinforcement from alcohol predate teens’ first drinking experiences, and predict drinking onset [85].

Direct experience may refute, confirm, or enhance pre-use expectancies. The learned expectations and experiences of specific outcomes as they occur with a specific appetitive behavior likely play an important role in addiction specificity. For example, heavier drinkers differ from light drinkers on activation of expectancies of positive arousing effects *versus* sedating effects of alcohol [80,86,87]. Additionally, research suggests that individuals with a single addictive behavior (e.g., alcohol only) differ from those who engage in multiple addictive behaviors (e.g., alcohol and marijuana) in the degree to which they hold positive expectations about the second behavior, suggesting the possibility for an uncoupling of expectations across addictive behaviors [87]. For example, there may be positivesedating marijuana use expectancies that would be inconsistent with positive-arousing alcohol use expectancies. Some persons may prefer one drug over the other due to these different expectancies, with a preference for sedation or arousal. Others may use both drugs with the expectation that they can use them to fluctuate or balance out their level of arousal [8]. Experiences with addictive behaviors thus may create subjective physiological expectancies that are addiction-specific.

Expectations associated with an addiction also may involve one’s perceptions of the social images (or general lifestyle characteristics) associated with participation in the behavior. For example, gambling or shopping addictions may be associated with social images of living luxuriously, love or sex addictions may be associated with social images of intimacy or social power, and marijuana addiction may be associated with living a countercultural lifestyle [e.g., 88]. In addition, perceptions of the gradient of reinforcement value functions portraying different addictive behaviors may vary in steepness, leading to selection of one addiction with a steeper gradient (more reinforcement value per unit time) over another [89]. Through any number of determinants of expectancy differentiation (e.g., mass media impact, family or peer social learning, experiences with an addictive behavior), social image expectations may take shape and impact addiction specificity. As an individual’s social activities begin to increasingly involve the addiction and other addicts or providers of the addiction, it may become possible to convince oneself that the addictive behavior does not interfere with and may even actually facilitate one’s daily activities. One may come to rely on a specific addiction, avoiding all others, if this addiction is perceived to meet many of one’s expectations for their life (e.g., there are people who might say that their life is “all right” as long as they have their marijuana).

## 3. Future Research Needs and Conclusions

While it is clear that addiction specificity is a phenomenon prevalent in the majority of the U.S. adult population who experience addiction problems [14], at least five research directions should be undertaken to better understand the parameters of addiction specificity or the PACE model as an explanatory device. First, hard epidemiologic data are lacking on addiction specificity [14]. That is, this empirical phenomenon is grossly understudied. Data need to be collected and examined across multiple addictive behaviors (and from diverse populations) to adequately assess the development of addiction specificity [14]. In particular, it would be informative to obtain data on the trajectories of addiction specificity. That is, data comparing the age of onset, duration, recurrence, or recovery from different constellations of addictions would be beneficial for understanding how different patterns of addiction specificity might occur as well as assist in clinical intervention development. It is plausible that the developmental trajectories for different addictions vary. For example, it is possible that addiction to exercise develops quite slowly because it can takes years for one to get in good enough shape to be able to exercise excessively. On the other hand, addiction to cigarette smoking may occur rather quickly. Different steepness in trajectories may provide one reason why more people may become addicted to one behavior (e.g., cigarette smoking) *versus* another (e.g., exercise). In addition, possibly, people who become addicted to a lower trajectory addiction (e.g., exercise) may become addicted to other lower trajectory addictions (e.g., workaholism), at least more so than persons who tend to become addicted to high trajectory addictions. Treatment implications may vary in terms of focus on fear of loss of slowly gained “expertise” (e.g., exercise and work) *versus* instruction in delaying gratification (e.g., drug use). Certainly, these ideas are speculative [90, 91].

Second, empirical evidence further supporting or refuting operation of the PACE variables on addiction specificity is necessary. In particular, research is needed to discern separability of the four dimensions of the PACE model, and the interplay between them (e.g., interaction effects) in an effort to better understand how, and to what degree each make a contribution to addiction specificity. Empirical testing of the PACE model will require psychometrically sound measures for each dimension, which are not yet available. However, we suggest that measures of pragmatics should quantify the degree of accessibility of the supply of the addictive agent, the perceived awareness of supply sources, the level of acquisition skill that an individual possesses or perceives possessing, and the efficacy of the means of exchange used in the pursuit of the addictive behavior. Measures of attraction should allow for the differentiation between individuals with high preferences for a specific behavior from those with low preference towards the behavior. For example, one might be asked on rating scales how much they like the social context of the addictive behavior, the rituals involved in engaging in the behavior, or the way the behavior feels. Measures of communication should assess familiarity with an addiction-specific language. For example, one might be asked how much slang pertaining to an addictive behavior they think they know, how much slang they know that nonparticipants in the behavior would be unlikely to know, or to what extent they tend to communicate differently with others who engage in the behavior *versus* those who don’t. The development of communication-type items is likely to require extensive qualitative and quantitative research to accurately gage communication regarding particular targets of addiction.

Expectation variables will need to be operationalized carefully to establish clear parameters that differentiate it from the other PACE variables. For example, one may be asked to what extent the behavior met their expectations, or how likely it was that the behavior would result in specific outcomes (e.g., degree to which the behavior helps one achieve a desired social image, fits well within one’s daily activities). Such items would need to be differentiated from ones, for example, that asked to what extent the behavior felt good, or was liked (aspects of the attraction variable). Possibly, items that request subjective probability information and, in particular, information that is relatively cognitive (*versus* affective) in nature, would best delineate the expectancy dimension. Figure 1 is an attempt to provide one means of conceptualizing how the PACE model components may relate to each other, but its function is heuristic at this point. Arguably, it is possible that overlap among some of the dimensions may exist and require some refinement in order to effectively differentiate the components (e.g., measurement of attraction *versus* expectations). This could potentially complicate understanding of the role each dimension serves in discriminating unique patterns of addiction.

Third, assuming the usefulness of separating the four dimensions, the operation of the PACE variables may differ in relative importance across different addictive behaviors, which may or may not reflect the reinforcement valence of these behaviors. For example, pragmatics may be a relatively important determinant of relatively hard-to-locate addictive behaviors (e.g., injection drug use, perhaps regular alcohol use among preteens), but may not be as important a determinant of easy-to-locate behaviors (e.g., eating, alcohol use among adults). Some addictions may be attractive to a relatively small percentage of the population (e.g., exercise), whereas other behaviors may have wide appeal (e.g., food). It is highly likely that each addiction is associated with specialized words or slang. However, is also possible that relatively socially acceptable addictive behaviors (eating, working, exercise) have fewer words associated with them to disguise their manifestations from nonparticipants. Finally, it is possible that different addictive behaviors are associated with different expectations (e.g., hedonism *versus* nurturance [23]). Examination of the relative importance of different PACE variables with different patterns of addiction specificity will require much work.

Fourth, while the current paper generally focused on addiction specificity from an individual differences perspective, it is important to note that there may be different patterns of addiction specificity within individuals over time. That is, when an addiction or finite set of addictions is terminated, a second addiction or set of addictions may or may not emerge. Longitudinal studies that assess the chronicity and/or fluidity of addictions within individuals will provide valuable information regarding how effectively the model delineates specificity *versus* co-occurrence. The PACE model as presented herein does not address the temporal stability of one’ addiction specificity propensity, but we nonetheless acknowledge that within-person variability in cross-addiction tendencies is certainly possible. This is an important issue that should be addressed in future work.

Finally, if the PACE model is useful in explaining addiction specificity then there also may be some clinical research implications. Understanding the association of PACE constituents with different individual addiction specificity trajectories may be useful for assessment research planning and treatment tailoring. For example, PACE information could be tested to potentially identify those who would benefit from interventions that target a single addictive behavior with a steep trajectory (e.g., nicotine replacement for tobacco) *versus* interventions which would be more useful for those prone to co-occurring addictions with a slower trajectory (e.g., learning new ways to manage anhedonia could benefit many different addictions).

In summary, addiction specificity may be a complementary concept to addiction co-occurrence, identifying reasons for non-overlap among different patterns of addictive behaviors. We propose a PACE model, which delineates pragmatics, attraction, communication and expectation components as being a useful framework for investigation on the determinants of addiction specificity. Further research applying the PACE model to addiction specificity may eventually yield clinical applications that help reduce the public health burden associated with addiction.
